# Supplementary material for: Multi-Echo Complex Quantitative Susceptibility Mapping and Quantitative Blood Oxygen Level-Dependent Magnitude (mcQSM + qBOLD or mcQQ) for Oxygen Extraction Fraction (OEF) Mapping
Source: Bioengineering (Basel). 2024 Jan 29;11(2):131. doi: 10.3390/bioengineering11020131 (PMC10886243; doi:10.3390/bioengineering11020131)
Supplement: Supplementary file 1 [file bioengineering-11-00131-s001.zip › bioengineering-2814521-supplementary.pdf]

Supplementary Materials

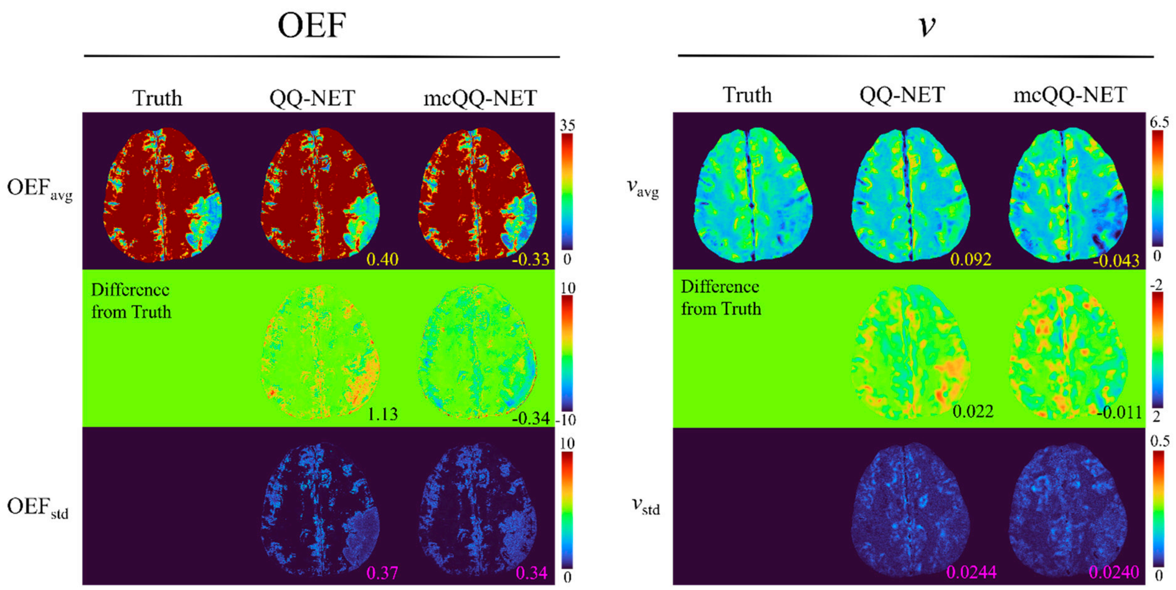

Figure S1. Comparison of OEF and v obtained by QQ-NET and mcQQ-NET in the simulated brain (Test Data 1). OEF_{avg} and v_{avg} indicate the average OEF and v maps, respectively, from five trials. OEF_{avg} and v_{avg} are shown in the unit of [%]. The numbers in yellow and black represent the mean error in the whole brain and lesion, respectively. QQ-NET showed higher OEF_{avg} and v_{avg} than the ground truth in low OEF lesions. Compared to QQ-NET, mcQQ-NET demonstrated a smaller bias in OEF_{avg} and v_{avg} in the lesion.

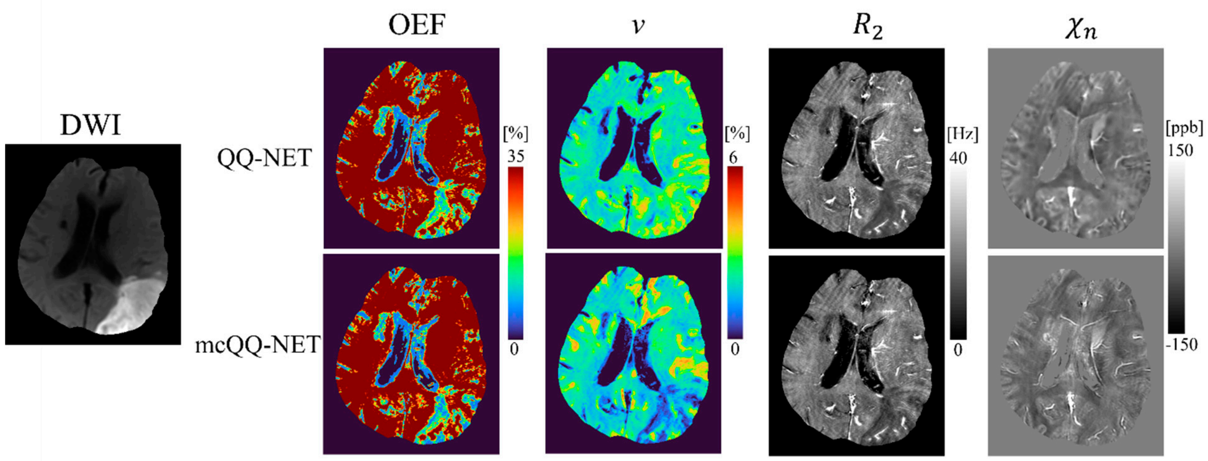

Figure S2. Comparison of OEF, v, R_2, and χ_n maps between QQ-NET and mcQQ-NET in a stroke patient imaged 4 days post stroke onset (Test Data 2). mcQQ-NET provided low OEF and low v values in the DWI-defined lesion.
